# Supplementary material for: Histone variant H2A.Z modulates nucleosome dynamics to promote DNA accessibility
Source: Nat Commun. 2023 Feb 11;14:769. doi: 10.1038/s41467-023-36465-5 (PMC9918499; doi:10.1038/s41467-023-36465-5)
Supplement: Supplementary file 1 — Supplementary Information [file 41467_2023_36465_MOESM1_ESM.pdf]

Histone variant H2A.Z modulates nucleosome dynamics to promote DNA accessibility

Shuxiang Li[1], Tiejun Wei[1] and Anna R. Panchenko[1,2,3]*

[1] Department of Pathology and Molecular Medicine, Queen's University, ON, Canada

[2] Department of Biology and Molecular Sciences, Queen's University, ON, Canada

[3] School of Computing, Queen's University, ON, Canada

*- corresponding author, anna.panchenko@queensu.ca

Construction of nucleosome structural models

Each simulation system in this study includes the nucleosome core particle and two straight 20 bp long DNA duplexes as linkers. To create a structural model of the full nucleosome with a native DNA sequence (*Homo sapiens TP53* gene), we first identified the precise translational positioning of DNA with respect to the histone octamer. To do this, we applied a previously developed nucleosome mapping protocol to the Micrococcal nuclease (MNase-seq) experimental data using the hg19 human genome assembly [1]. We selected fragments of 147 base pair lengths as the nucleosomal DNA length. The dyad positions were determined as the middle points of all 147 bp fragments [2]. Then a 15-bp tri-weight kernel function was used to smooth the mid-fragment counts with bwtool [3], and we obtained the dyad positions with local maximum values of the smoothed counts. After that, the +1 nucleosome was identified as the first nucleosome located downstream of the transcription start site of the *TP53* gene. The native DNA sequence (187 bp) of the +1 nucleosome is shown in Supplementary Figure 2.

The high-resolution X-ray structure of a nucleosome core particle (PDB ID: 3AFA) was used to build the initial structural model of the canonical H2A ($NUC_{H2A/H2A}$). The 20 bp linker DNA flanking the core particle on each side linker DNA was built by linearly adding 20 bp DNA segments from both ends using the NAB software [4]. The native gene sequence (Supplementary Figure 2) was embedded into this structural model using the 3DNA program [5]. The missing residues of the histone tails were modeled by linearly extending the existing tail conformations by Chimera [6]. The dihedral angles for each residue in the histone tails were assigned with Φ angle = −60° and Ψ angle = 30°. This modeling protocol was also applied to construct the nucleosome models of $NUC_{H2A.Z/H2A.Z}$, $NUC_{H2A.Z/H2A.Z+H3.3/H3.3}$ and $NUC_{H2A/H2A.Z+H3/H3.3.}$ Note that PDB IDs 1F66, 5B33 and 5B32 which contain different combinations of human histone variants H2A.Z.1 and H3.3 were used to build the models of the $NUC_{H2A.Z/H2A.Z}$, $NUC_{H2A.Z/H2A.Z+H3.3/H3.3}$ and $NUC_{H2A/H2A.Z+H3/H3.3}$, respectively.

The above-built initial structural models of $NUC_{H2A/H2A}$ and $NUC_{H2A.Z/H2A.Z}$ were used to construct the tail-swapped models of $H2A_{H2AZtail}$ (two copies of H2A with H2A.Z C-terminal tail) and $H2AZ_{H2Atail}$ (two copies of H2A.Z with H2A C-terminal tail). To accomplish this, the H2A C-terminal tail (KTESHHKAKGK, residue index 120-130) of the $NUC_{H2A/H2A}$ was truncated and then modeled by adding amino acids of KGQQKTV (H2A.Z C-terminal tail) with Chimera [6]. This new model is referred to as $H2A_{H2AZCtail}$. Similarly, the H2A.Z C-terminal tail (KGQQKTV, residue index 122-128) of the $NUC_{H2A.Z/H2A.Z}$ was truncated and then modeled by adding amino acids of KTESHHKAKGK (H2A C-terminal tail). This new model is referred to as $H2AZ_{H2Atail}$.

Supplementary Table 1. Summary of main simulation systems and simulation setup.

Simulation systems	Force field	Water model	Simulation time
**$NUC_{H2A/H2A}$ (H2A/H2A+H3/H3)**	AMBER FF14SB + OL15	OPC	3 runs * 7000ns
**$NUC_{H2A.Z/H2A.Z}$ (H2A.Z/H2A.Z+H3/H3)**	AMBER FF14SB + OL15	OPC	3 runs * 7000ns
**$NUC_{H2A.Z/H2A.Z+H3.3/H3.3}$ (H2A.Z/H2A.Z+H3.3/H3.3)**	AMBER FF14SB + OL15	OPC	3 runs * 7000ns
**$NUC_{H2A/H2A.Z+H3/H3.3}$ (H2A/H2A.Z+H3/H3.3)**	AMBER FF14SB + OL15	OPC	3 runs * 7000ns

Supplementary Table 2. Summary of supplementary simulation systems.

Simulation systems	Force field	Water model	Simulation time
**$H2A_{H2AZtail}$ (two copies of H2A with H2A.Z C-terminal tail)**	AMBER FF14SB + OL15	OPC	1 run * 2000ns
**$H2AZ_{H2Atail}$ (two copies of H2A.Z with H2A C-terminal tail)**	AMBER FF14SB + OL15	OPC	1 run * 2000ns
**$H2AZ_{restraint}$**	AMBER FF14SB + OL15	OPC	1 run * 2000ns

Supplementary Table 3. Summary of simulation systems, volume, number of particles and simulation performance.

Simulation system	Volume (nm^3)	Total atoms	HPC hardware	Performance (ns/day)	Simulation time	Time required (days)
**$NUC_{H2A/H2A}$**	20485.1	2,688,727	CPU: IBM Power9 GPU: 5x NVIDIA V100-SMX2	18.6	7000 ns	~376
**$NUC_{H2A.Z/H2A.Z}$**	20485.1	2,688,887		18.6	7000 ns	~376
**$NUC_{H2A.Z/H2A.Z+H3.3/H3.3}$**	20485.1	2,688,909		18.6	7000 ns	~376
**$NUC_{H2A/H2A.Z+H3/H3.3}$**	20485.1	2,688,768		18.6	7000 ns	~376
**$H2A_{H2AZtail}$**	20485.1	2,688,721	CPU: IBM Power9 GPU: 10 x NVIDIA V100-SMX2	27.4	2000 ns	~73
**$H2AZ_{H2Atail}$**	20485.1	2,688,781		27.4	2000 ns	~73
**$H2AZ_{restraint}$**	20485.1	2,688,887		27.4	2000 ns	~73

Supplementary Table 4. Binding free energy of association between histone octamer and DNA by MM/GBSA analysis. The calculation was performed using the first and last 200 ns trajectories. For each histone type, the standard error (SE) of the mean from three independent simulation runs for two histone copies was estimated (n=6). Lower values of ΔG coreespond to more stable structures. No considerable DNA unwrapping was observed during the first and last 200ns.

Simulation system	Histone type	ΔG (kcal/mol) first 200ns	ΔG (kcal/mol) last 200ns
$NUC_{H2A/H2A}$	H3	-230.0 ± 19.7	-264.2 ± 26.5
	H4	-111.2 ± 3.9	-127.1 ± 12.0
	H2A	-124.9 ± 8.5	-135.1 ± 7.4
	H2B	-113.3 ± 17.0	-121.2 ± 8.9
$NUC_{H2A.Z/H2A.Z}$	H3	-233.8 ± 11.0	-240.1 ± 33.8
	H4	-118.7 ± 10.3	-133.0 ± 11.1
	H2A.Z	-93.1 ± 13.0	-106.5 ± 16.9
	H2B	-91.7 ± 7.1	-97.3 ± 10.9

Supplementary Figure 1. Cartoon representations of an initial nucleosome model in the MD simulations. a) $NUC_{H2A/H2A}$ system. b) $NUC_{H2A.Z/H2A.Z}$ system. c) $NUC_{H2A.Z/H2A.Z+H3.3/H3.3}$ system. d) $NUC_{H2A/H2A.Z+H3/H3.3}$ system.

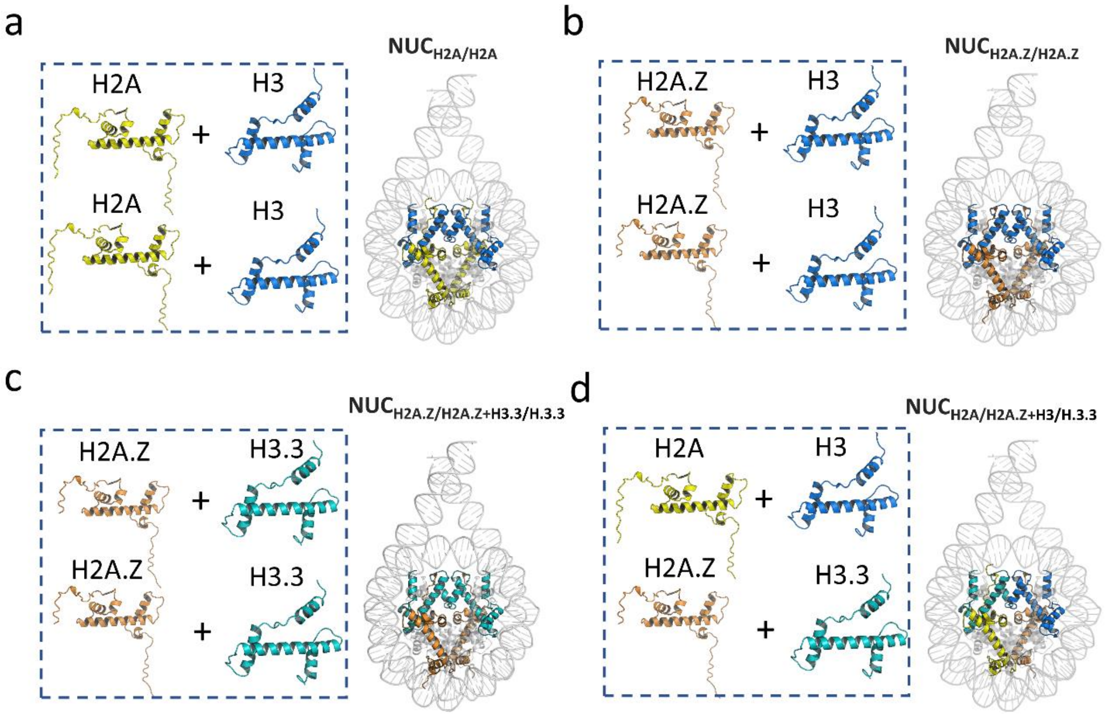

Supplementary Figure 2. *Homo sapiens TP53* gene sequence (5’ to 3’) was used to construct all initial nucleosome models. The DNA sequence is labeled based on the relative position with respect to the dyad. The dyad position is shown in red.

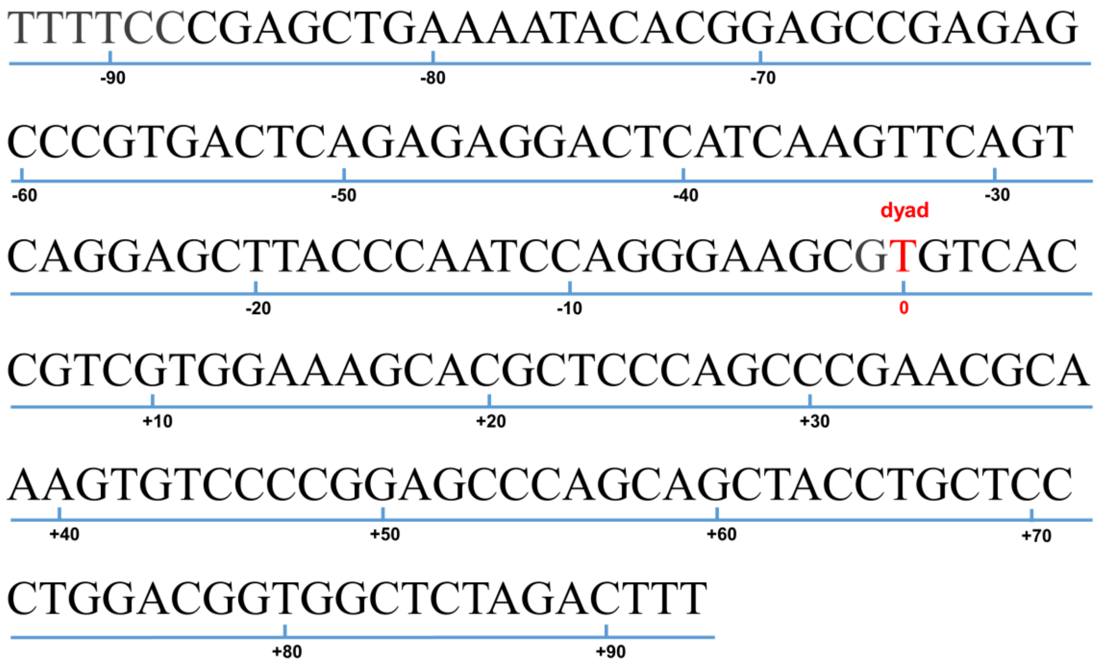

Supplementary Figure 3. Dynamics of nucleosomal and linker DNA. a), b) and c) show 2D projections of DNA conformations for the $NUC_{H2A.Z/H2A.Z}$ system in each simulation run. d), e) and f) show 2D projections of DNA conformations for the $NUC_{H2A/H2A}$ system in each simulation run.

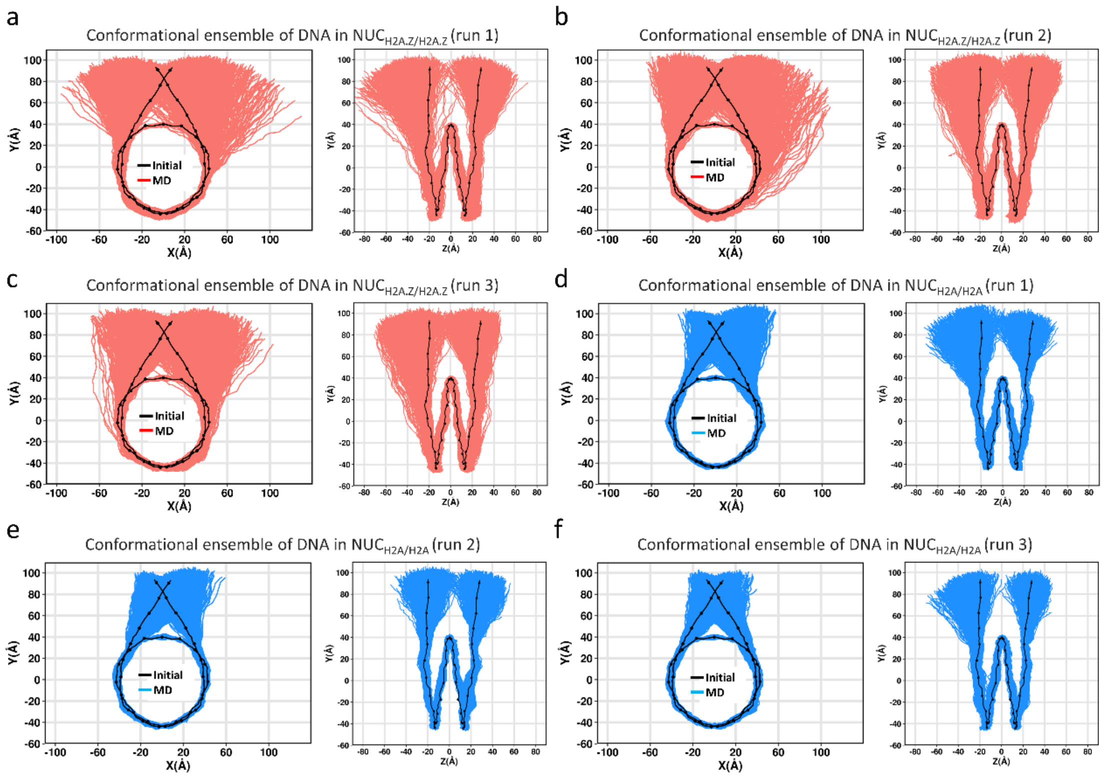

Supplementary Figure 4. Dynamics of the nucleosomal and linker DNA. a) 2D projections of DNA conformations for $NUC_{H2A.Z/H2A.Z+H3.3/H3.3}$ from three independent simulation runs. b) Same as a) but for $NUC_{H2A/H2A.Z+H3/H3.3}$ nucleosomes. c) Distributions of a total number of unwrapped base pairs from entry and exit DNA sides, see Supplementary Figure 5 for the definitions of DNA regions.

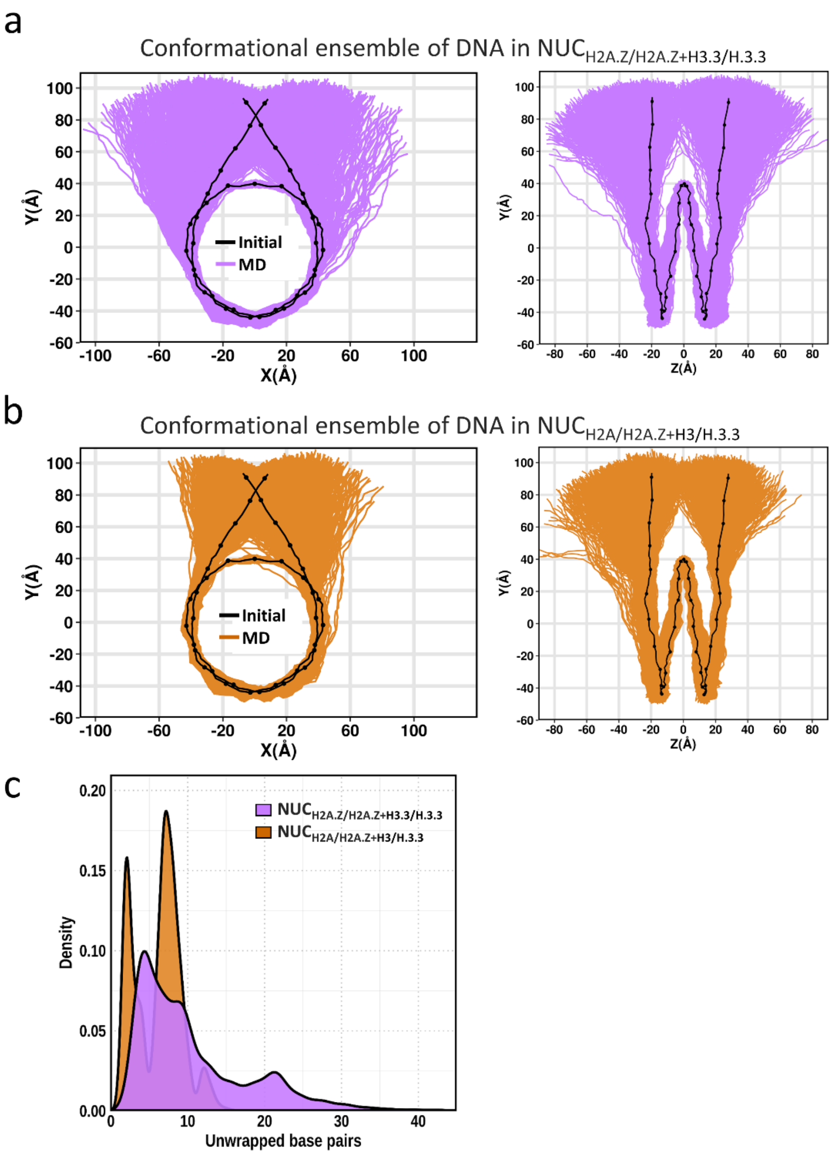

Supplementary Figure 5. Definitions of DNA regions based on their relative positions with respect to the dyad (base-pair index “0”).

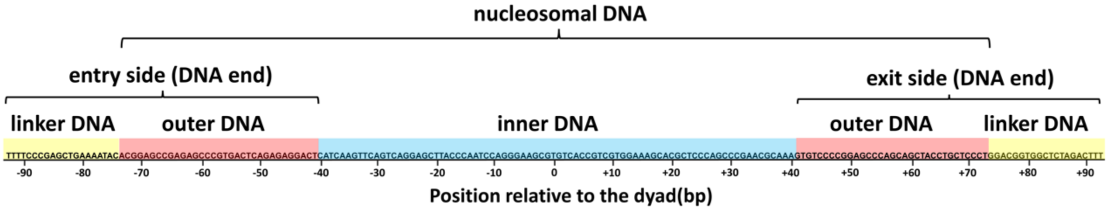

Supplementary Figure 6. The time evolution of the H2A.Z C-terminal tail - DNA contacts and the total number of unwrapped base pairs from both DNA ends in $NUC_{H2A.Z/H2A.Z}$ nucleosomes from three independent simulation runs. a) Simulation run 1 of $NUC_{H2A.Z/H2A.Z}$. b) Simulation run 2 of $NUC_{H2A.Z/H2A.Z}$. c) Simulation run 3 of $NUC_{H2A.Z/H2A.Z}$. The number of contacts of H2A.Z C-terminal tail with total DNA, outer DNA and inner DNA regions are shown in blue, green and purple, respectively. The lines with faded colors correspond to raw data and are smoothed with a Savitzky-Golay filter using a ten ns window and first-degree polynomial (dark color lines).

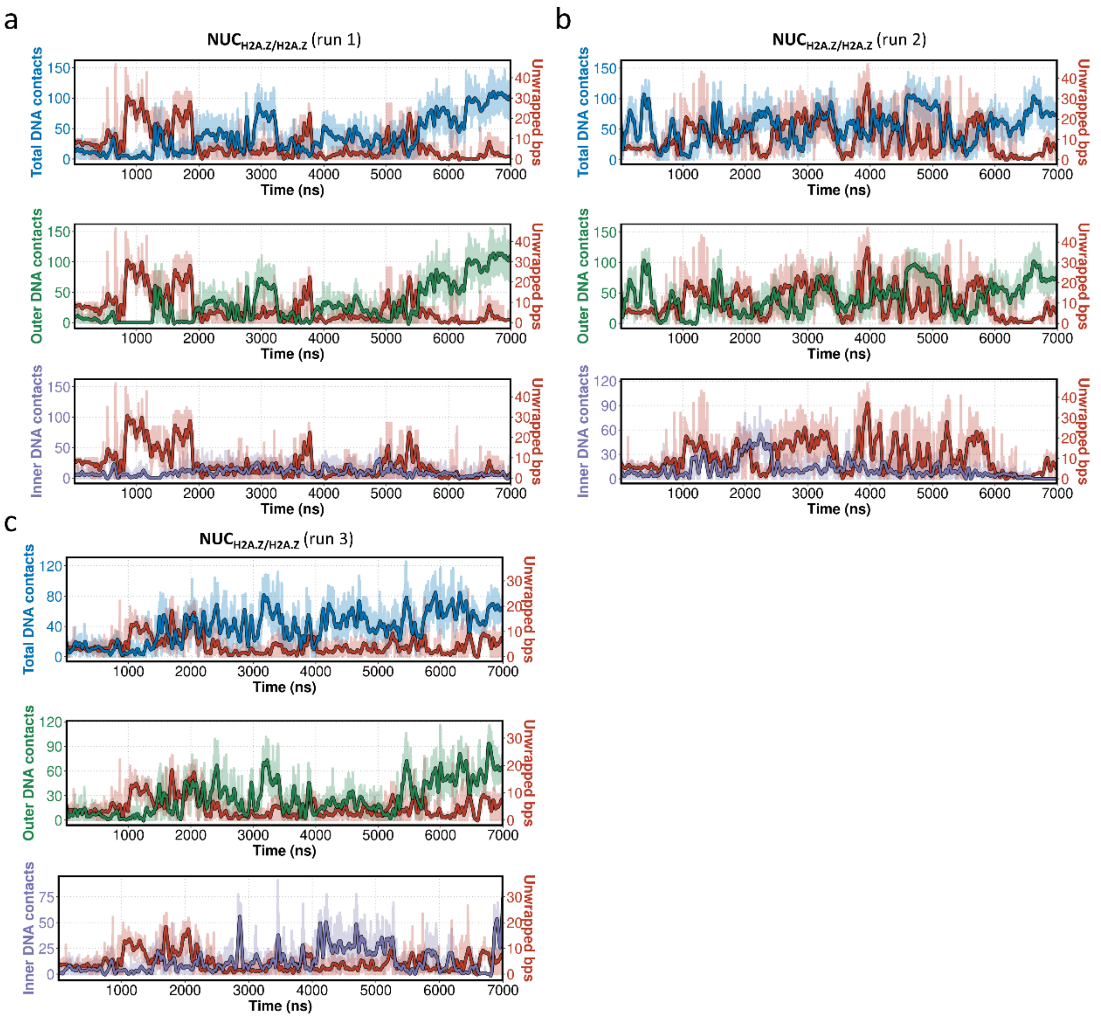

Supplementary Figure 7. The time evolution of the H2A C-terminal tail - DNA contacts and the total number of unwrapped base pairs from both DNA ends in $NUC_{H2A/H2A}$ nucleosome from three independent simulation runs. a) Simulation run 1 of $NUC_{H2A/H2A}$. b) Simulation run 2 of $NUC_{H2A/H2A}$. c) Simulation run 3 of $NUC_{H2A/H2A}$. The number of contacts of H2A.Z C-terminal tail with total DNA, outer DNA and inner DNA regions are shown in blue, green and purple, respectively. The lines with faded colors correspond to raw data and are smoothed with a Savitzky-Golay filter using a ten ns window and first-degree polynomial (dark color lines).

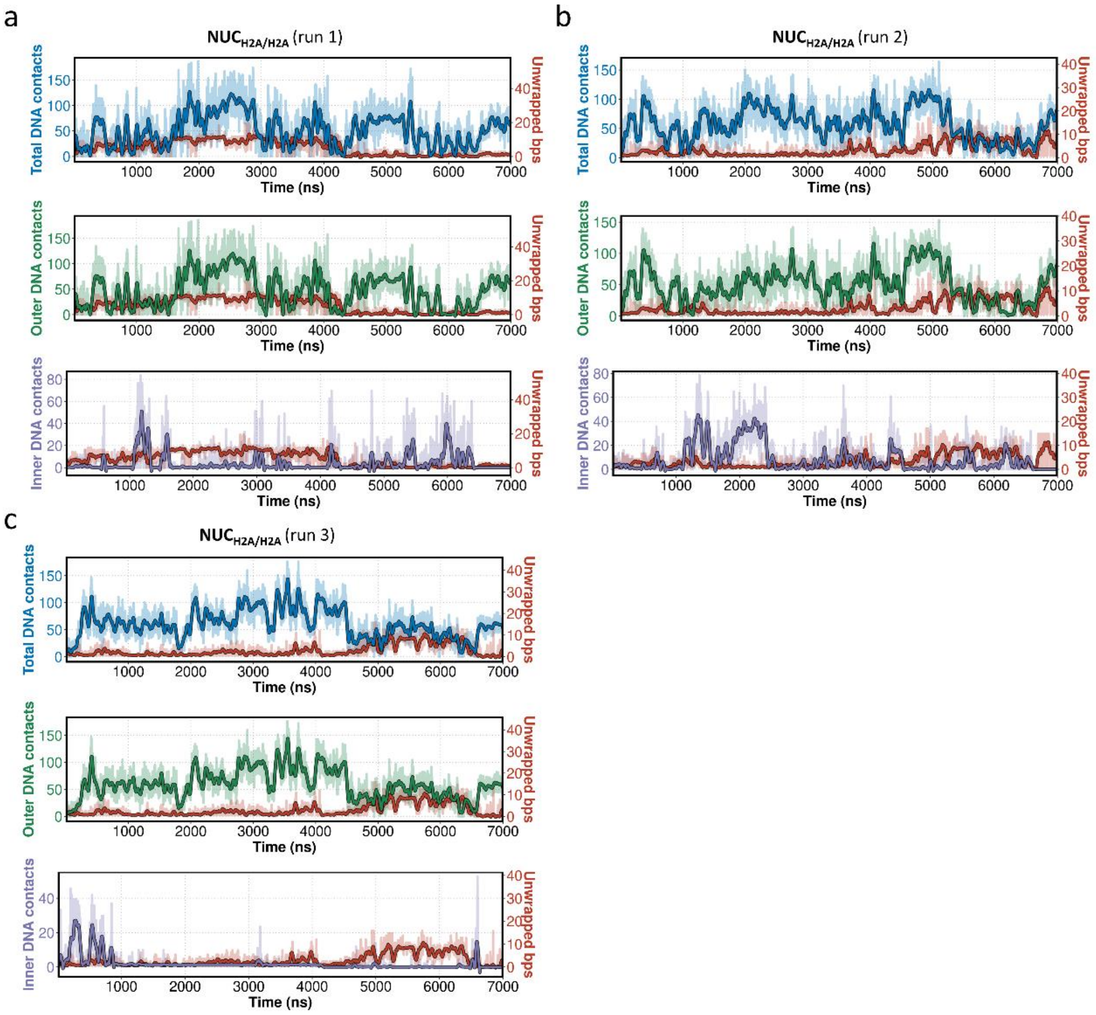

Supplementary Figure 8. Cartoon representations of the initial nucleosome models by swapping the H2A and H2A.Z C-terminal tails. a) $H2A_{H2AZtail}$ system contains two copies of H2A (yellow) with H2A.Z C-terminal tails (oragne). b) $H2AZ_{H2Atail}$ system contains two copies of H2A.Z (orange) with H2A.Z C-terminal tails (yellow).

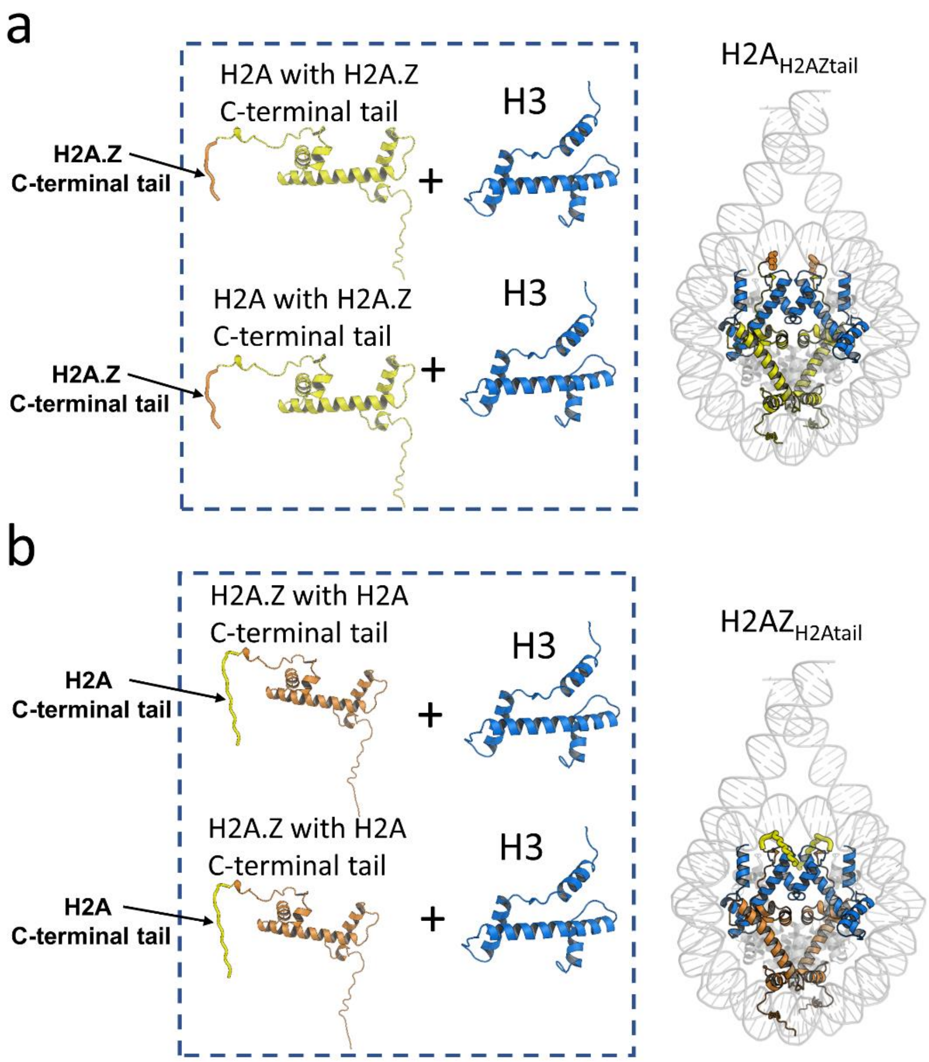

Supplementary Figure 9. Dynamics of systems with swapped tails. a) 2D projections of DNA conformations for $H2A_{H2AZtail}$ nucleosome. b) 2D projections of DNA conformations for $H2AZ_{H2Atail}$ nucleosome. c) Distributions of a total number of unwrapped base pairs of $H2A_{H2AZtail}$ (blue) and $H2AZ_{H2Atail}$ (red) on 2 microseconds time scale. d) Distributions of a total number of unwrapped base pairs of $NUC_{H2A/H2A}$ (blue) and $NUC_{H2A.Z/H2A.Z}$ (orange) nucleosomes on 2 microsecond time scale from three independent simulation runs (solid, dotted and dashed lines).

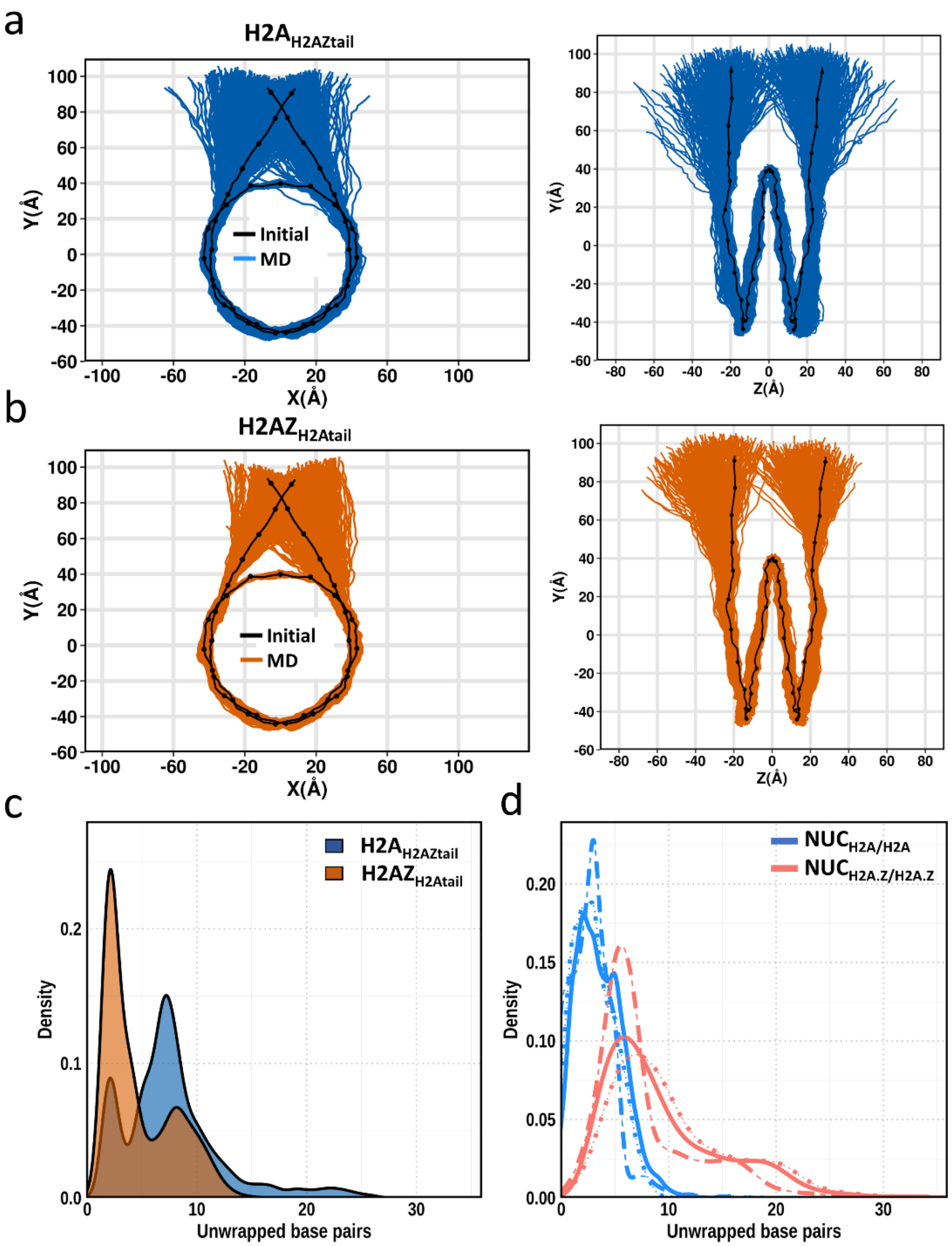

Supplementary Figure 10. H2A.Z C-terminal tail may modulate the nucleosome array compaction. a) Cryo-EM structures of chromatin fibers containing H2A.Z nucleosomes 7. b) X-ray structure of a tetranucleosome 8. c) A representative H2A.Z C-terminal tail conformation (red) from $NUC_{H2A.Z/H2A.Z}$ with the extended tail conformation from our simulations.

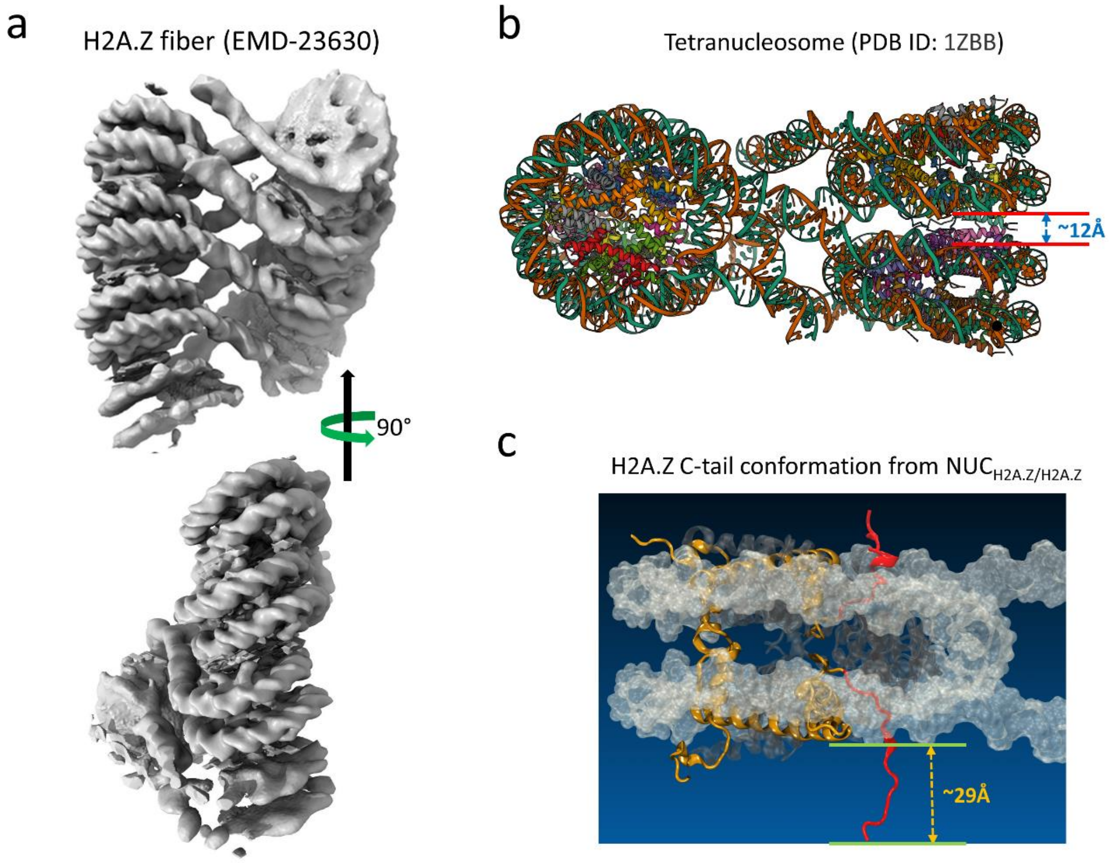

Supplementary Figure 11. a) The values of root-mean square fluctuations (RMSF) for Cα atoms of histone H3 globular core domain in $NUC_{H2A/H2A}$ nucleosome (blue) and H2A.Z in $NUC_{H2A.Z/H2A.Z}$ nucleosome (red). The error bars represent standard errors of RMSF (n= 6) calculated from three independent simulation runs for two copies o H3. b) Same as a) but for histone H4 globular core domain.

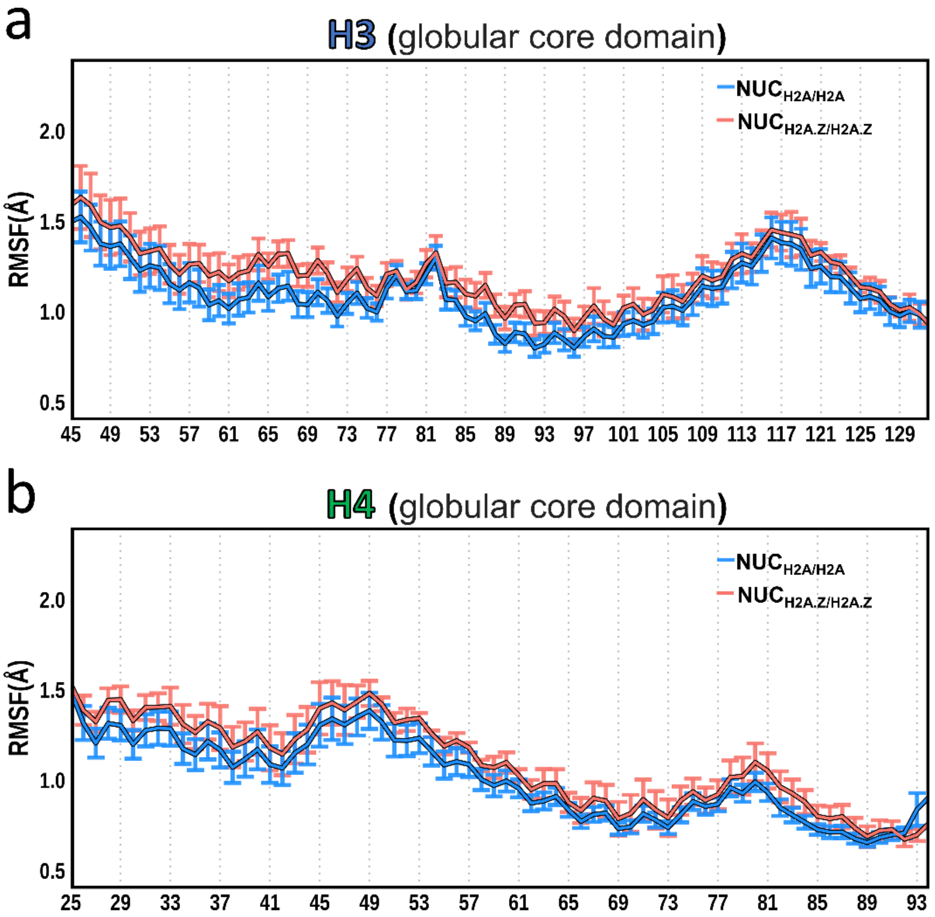

Supplementary Figure 12. Plasticity of the histone octamer. a) The conformational ensemble of histone helices for H2A (blue) and other histones (grey) in $NUC_{H2A/H2A}$ nucleosome. b) The conformational ensemble of histone helices for H2A.Z (red) and other histones (grey) in $NUC_{H2A.Z/H2A.Z}$ nucleosome.

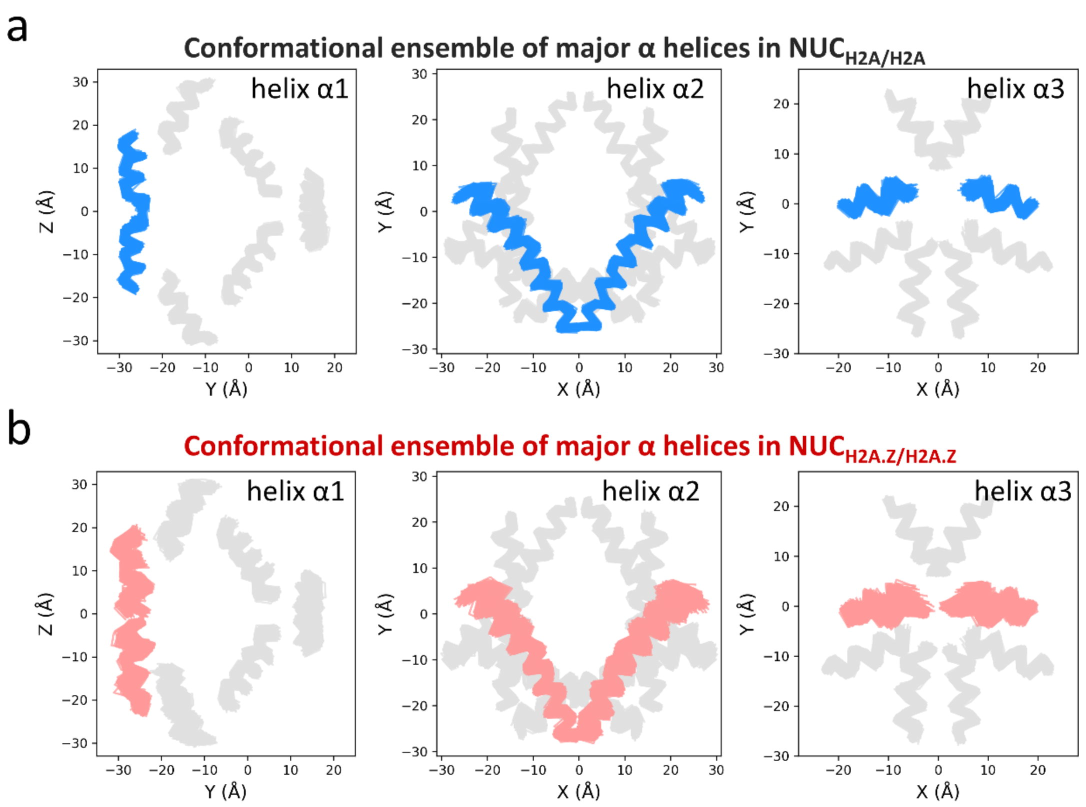

Supplementary Figure 13. Dynamics of H2A.Z nucleosome with restraints. a) 2D projections of DNA conformations for $H2AZ_{restraint}$ nucleosome. b) Distributions of a total number of unwrapped base pairs of $H2AZ_{restraint}$ on 2 microseconds time scale. c) Distributions of a total number of unwrapped base pairs of $NUC_{H2A.Z/H2A.Z}$ nucleosomes on 2 microsecond time scale from three independent simulation runs (solid, dotted and dashed lines).

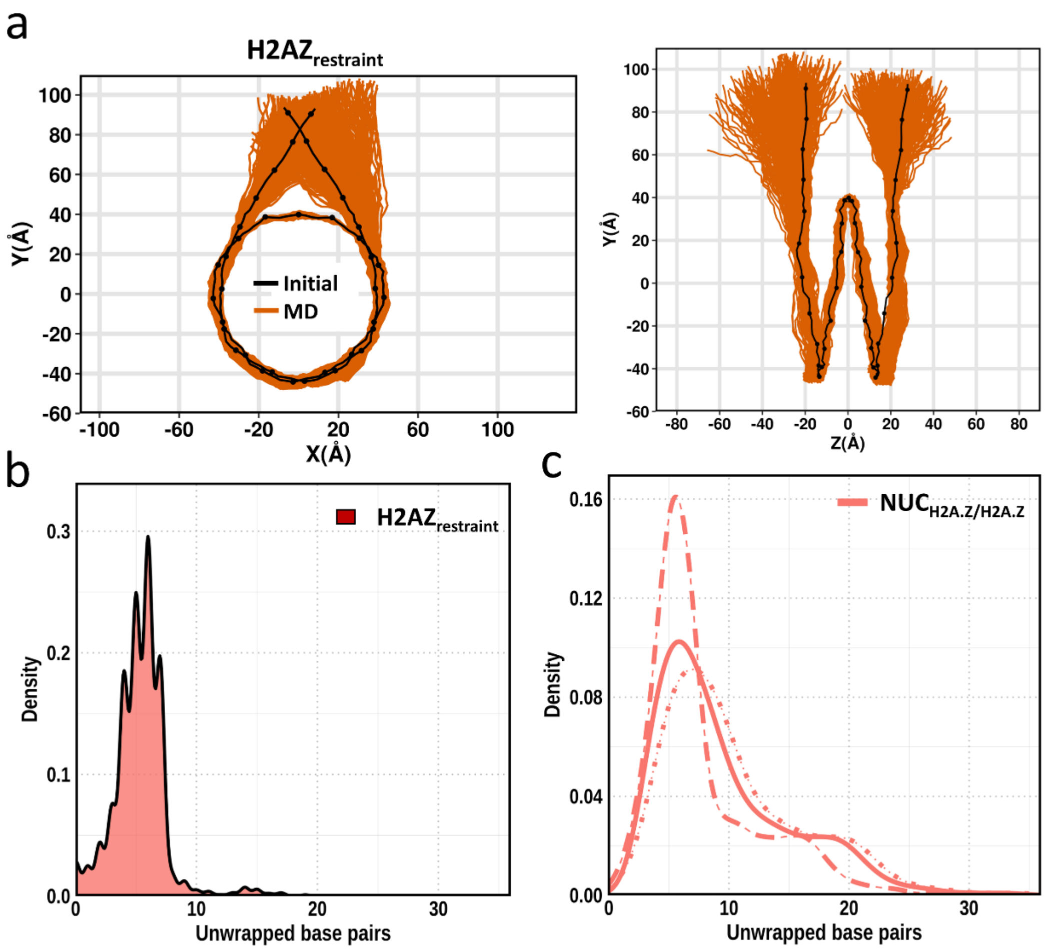

Supplementary Figure 14. a)The RMSF values for Cα atoms of histone H2A globular core domain in $H2A_{H2AZtail}$ nucleosome (blue) and H2A.Z in $H2AZ_{H2Atail}$ nucleosome (red). Each copy of H2A or H2A.Z is represented by solid and dashed lines, respectively. b) Same as a) but for histone H2B globular core domain.

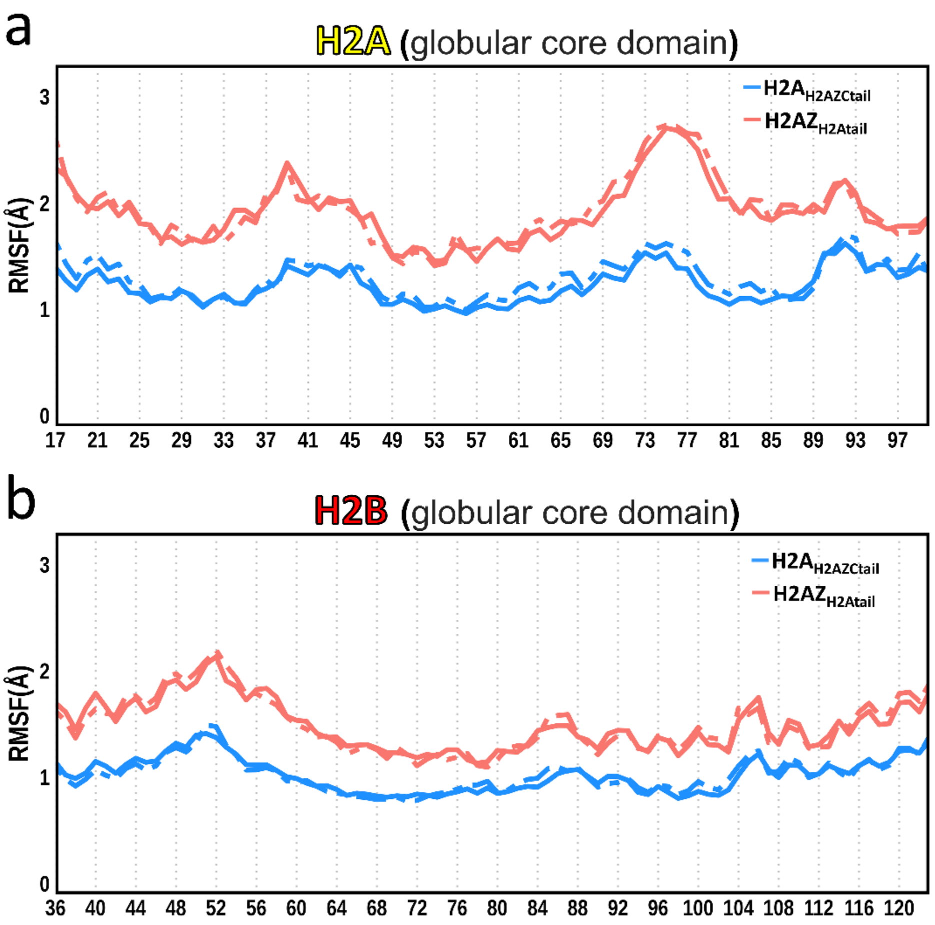

Supplementary Figure 15. Time evolution of the H2A.Z N-terminal tail - DNA contacts (green) and nucleosome gapping distances (orange) in the $NUC_{H2A.Z/H2A.Z}$ system from three simulation runs. a) Simulation run 1 of $NUC_{H2A.Z/H2A.Z}$. b) Simulation run 2 of $NUC_{H2A.Z/H2A.Z}$. c) Simulation run 3 of $NUC_{H2A.Z/H2A.Z}$.

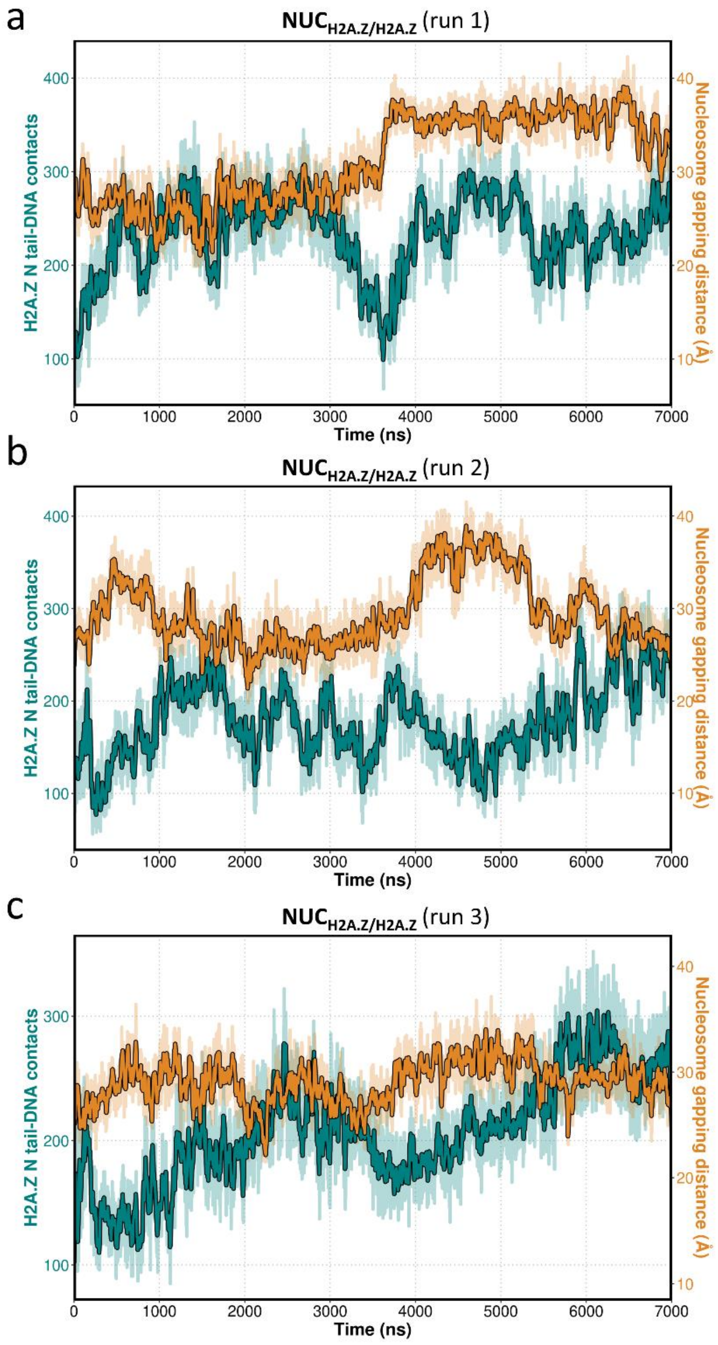

Supplementary Figure 16. Time evolution of the H2A N-terminal tail - DNA contacts (green) and nucleosome gapping distances (orange) in the $NUC_{H2A/H2A}$ system from three simulation runs. a) Simulation run 1 of $NUC_{H2A/H2A}$. b) Simulation run 2 of $NUC_{H2A/H2A}$. c) Simulation run 3 of $NUC_{H2A/H2A}$.

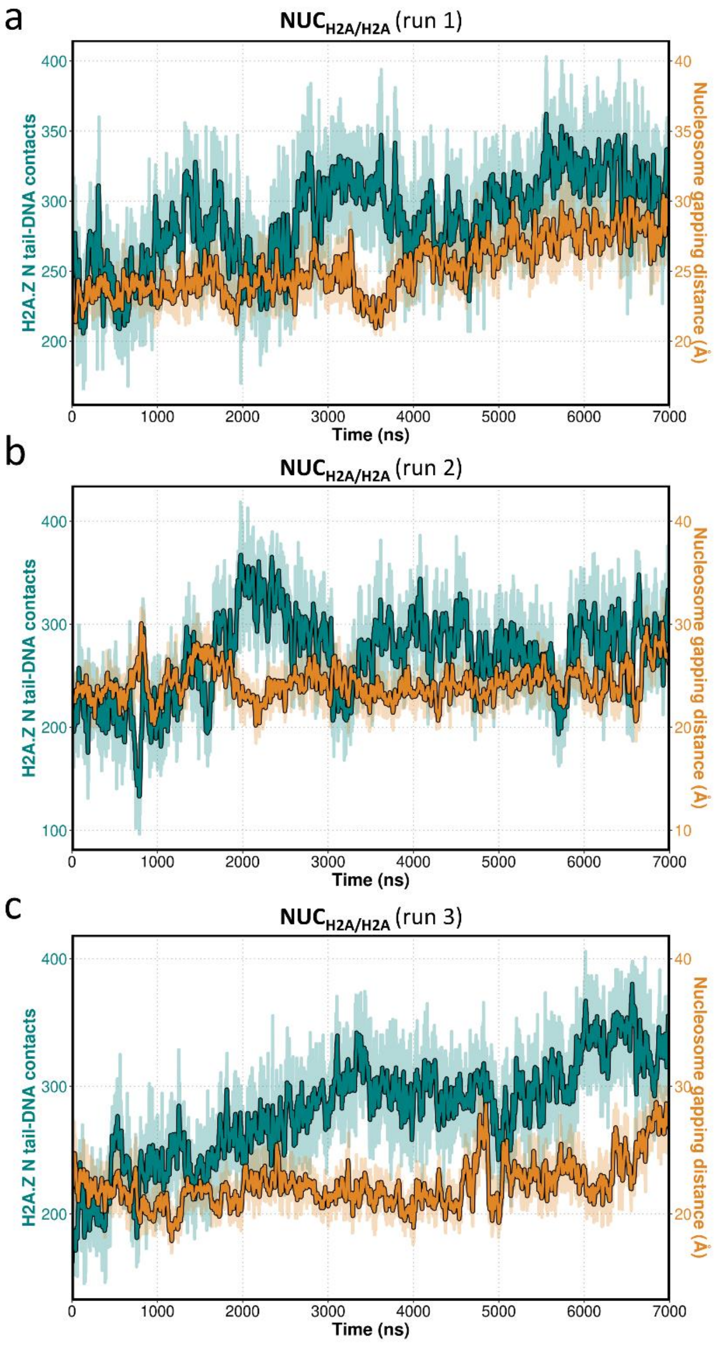

Supplementary Figure 17. a) Distributions of nucleosome gaping distances in H2AZ$_{restraint}$ system on 2 microsecond time scale. b) Distributions of nucleosome gaping distances in NUC$_{H2A/H2A}$ (blue) and NUC$_{H2A.Z/H2A.Z}$ (red) systems on 2 microsecond time scale from three independent simulation runs (solid, dotted and dashed lines).

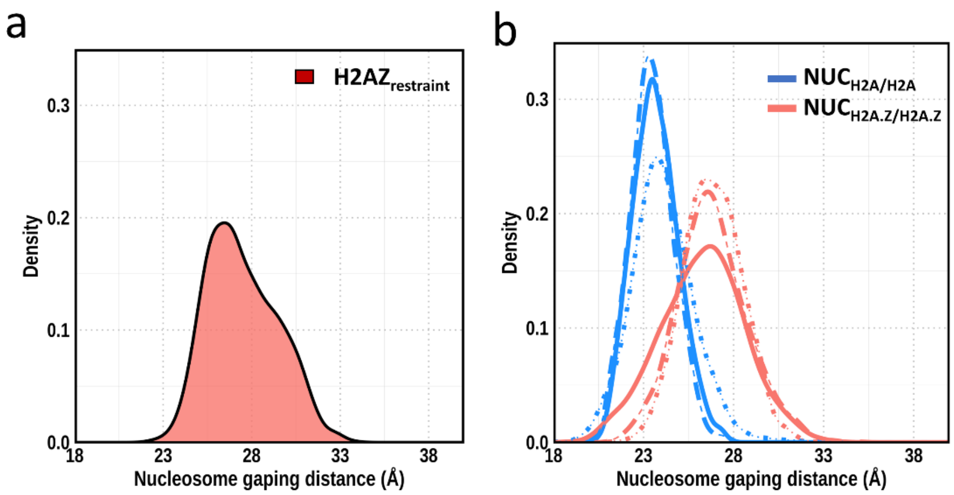

References

1. Pich, O., Muinos, F., Sabarinathan, R., Reyes-Salazar, I., Gonzalez-Perez, A. & Lopez-Bigas, N. Somatic and Germline Mutation Periodicity Follow the Orientation of the DNA Minor Groove around Nucleosomes. *Cell* **175**, 1074-1087 (2018).
2. Gaffney, D.J. et al. Controls of Nucleosome Positioning in the Human Genome. *PLoS Genet.* **8**, e1003036 (2012).
3. Pohl, A. & Beato, M. bwtool: a tool for bigWig files. *Bioinformatics* **30**, 1618-1619 (2014).
4. Salomon-Ferrer, R., Case, D.A. & Walker, R.C. An overview of the Amber biomolecular simulation package. *Wiley Interdiscip. Rev. Comput. Mol. Sci.* **3**, 198-210 (2013).
5. Li, S.X., Olson, W.K. & Lu, X.J. Web 3DNA 2.0 for the analysis, visualization, and modeling of 3D nucleic acid structures. *Nucleic Acids Res.* **47**, W26-W34 (2019).
6. Goddard, T.D. et al. UCSF ChimeraX: Meeting modern challenges in visualization and analysis. *Protein Sci.* **27**, 14-25 (2018).
7. Lewis, T.S., Sokolova, V., Jung, H., Ng, H. & Tan, D.Y. Structural basis of chromatin regulation by histone variant H2A.Z. *Nucleic Acids Res.* **49**, 11379-11391 (2021).
8. Schalch, T., Duda, S., Sargent, D.F. & Richmond, T.J. X-ray structure of a tetranucleosome and its implications for the chromatin fibre. *Nature* **436**, 138-141 (2005).
