# Supplementary material for: Histone variant H2A.Z modulates nucleosome dynamics to promote DNA accessibility
Source: Nat Commun. 2023 Feb 11;14:769. doi: 10.1038/s41467-023-36465-5 (PMC9918499; doi:10.1038/s41467-023-36465-5)
Supplement: Supplementary file 3 — Reporting Summary [file 41467_2023_36465_MOESM3_ESM.pdf]

nature portfolio

Corresponding author(s): Anna Panchenko

Last updated by author(s): Jan 24, 2023

Reporting Summary

Nature Portfolio wishes to improve the reproducibility of the work that we publish. This form provides structure for consistency and transparency in reporting. For further information on Nature Portfolio policies, see our Editorial Policies and the Editorial Policy Checklist.

Statistics

For all statistical analyses, confirm that the following items are present in the figure legend, table legend, main text, or Methods section.

n/a	Confirmed	
☐	☒	The exact sample size (*n*) for each experimental group/condition, given as a discrete number and unit of measurement
☐	☒	A statement on whether measurements were taken from distinct samples or whether the same sample was measured repeatedly
☒	☐	The statistical test(s) used AND whether they are one- or two-sided<br>*Only common tests should be described solely by name; describe more complex techniques in the Methods section.*
☒	☐	A description of all covariates tested
☒	☐	A description of any assumptions or corrections, such as tests of normality and adjustment for multiple comparisons
☐	☒	A full description of the statistical parameters including central tendency (e.g. means) or other basic estimates (e.g. regression coefficient) AND variation (e.g. standard deviation) or associated estimates of uncertainty (e.g. confidence intervals)
☒	☐	For null hypothesis testing, the test statistic (e.g. *F*, *t*, *r*) with confidence intervals, effect sizes, degrees of freedom and *P* value noted<br>*Give P values as exact values whenever suitable.*
☒	☐	For Bayesian analysis, information on the choice of priors and Markov chain Monte Carlo settings
☒	☐	For hierarchical and complex designs, identification of the appropriate level for tests and full reporting of outcomes
☒	☐	Estimates of effect sizes (e.g. Cohen's *d*, Pearson's *r*), indicating how they were calculated

Our web collection on statistics for biologists contains articles on many of the points above.

Software and code

Policy information about availability of computer code

Data collection	All molecular dynamic simulations were performed using GROMACS version 2020.6 (https://manual.gromacs.org/2020.6/install-guide/index.html)
Data analysis	VMD version 1.9.3 available at https://www.ks.uiuc.edu/Research/vmd/.<br>In-house python (version 3.7.4) codes are developed using the following key libraries: numpy1.19.2, pandas 1.3.4, vmd-python 3.0.6.<br>UCSF Chimera version 1.16 available at ttps://www.cgl.ucsf.edu/chimera/download.html.<br>Amber20 Package available at https://ambermd.org/AmberMD.php.<br>3DNA version 2.4 available at https://x3dna.org.<br>All the scripts are available at https://github.com/Panchenko-Lab/Supplementary_data_Li_et_al_2022

For manuscripts utilizing custom algorithms or software that are central to the research but not yet described in published literature, software must be made available to editors and reviewers. We strongly encourage code deposition in a community repository (e.g. GitHub). See the Nature Portfolio guidelines for submitting code & software for further information.

Data

Policy information about availability of data

All manuscripts must include a data availability statement. This statement should provide the following information, where applicable:

- Accession codes, unique identifiers, or web links for publicly available datasets
- A description of any restrictions on data availability
- For clinical datasets or third party data, please ensure that the statement adheres to our policy

The source data in this study have been deposited in GitHub at https://github.com/Panchenko-Lab/Supplementary_data_Li_et_al_2022. The molecular dynamics simulation trajectory data is provided in the Figshare repository at https://doi.org/10.6084/m9.figshare.21782570. Source data are provided with this paper. Nucleosome structures used in this study include PDBID 3AFA (https://www.rcsb.org/structure/3AFA), 1F66 (https://www.rcsb.org/structure/1F66), 5B33 (https://www.rcsb.org/structure/5B33) and 5B32 (https://www.rcsb.org/structure/5B32). The hg19 human genome assembly is available from https://www.ncbi.nlm.nih.gov/assembly/GCF_000001405.13/.

Human research participants

Policy information about studies involving human research participants and Sex and Gender in Research.

Reporting on sex and gender	*Use the terms sex (biological attribute) and gender (shaped by social and cultural circumstances) carefully in order to avoid confusing both terms. Indicate if findings apply to only one sex or gender; describe whether sex and gender were considered in study design whether sex and/or gender was determined based on self-reporting or assigned and methods used. Provide in the source data disaggregated sex and gender data where this information has been collected, and consent has been obtained for sharing of individual-level data; provide overall numbers in this Reporting Summary. Please state if this information has not been collected. Report sex- and gender-based analyses where performed, justify reasons for lack of sex- and gender-based analysis.*
Population characteristics	*Describe the covariate-relevant population characteristics of the human research participants (e.g. age, genotypic information, past and current diagnosis and treatment categories). If you filled out the behavioural & social sciences study design questions and have nothing to add here, write "See above."*
Recruitment	*Describe how participants were recruited. Outline any potential self-selection bias or other biases that may be present and how these are likely to impact results.*
Ethics oversight	*Identify the organization(s) that approved the study protocol.*

Note that full information on the approval of the study protocol must also be provided in the manuscript.

Field-specific reporting

Please select the one below that is the best fit for your research. If you are not sure, read the appropriate sections before making your selection.

☒ Life sciences ☐ Behavioural & social sciences ☐ Ecological, evolutionary & environmental sciences

For a reference copy of the document with all sections, see nature.com/documents/nr-reporting-summary-flat.pdf

Life sciences study design

All studies must disclose on these points even when the disclosure is negative.

Sample size	The sample size in molecular dynamics simulation studies is determined by the convergence of the simulation runs and variances of measurements. In this study, we performed simulations for four systems with three runs for each system. Each simulation run contains 70,000 frames for the simulated system. Our results show that calculated measurements such as the number of unwrapped DNA base pairs and the DNA-histone contacts vary little among different independent runs. These observations indicate the sufficient sample size of our studies.
Data exclusions	No data was excluded.
Replication	For each nucleosome system, we performed three independent simulation runs. All simulation runs are successfully performed and the calculated measurements vary little among different independent runs.
Randomization	In our molecular dynamics simulation studies, the entire frames (70,000 conformations) from each simulation trajectory were used for our data analysis.
Blinding	We do not collect any statistical samples in our simulation studies as all our judgments are based on the convergence of the simulation runs and variances of measurements among different runs. So blinding is not relevant to our study. The data from the simulation trajectories were directly used for analysis.

Reporting for specific materials, systems and methods

We require information from authors about some types of materials, experimental systems and methods used in many studies. Here, indicate whether each material, system or method listed is relevant to your study. If you are not sure if a list item applies to your research, read the appropriate section before selecting a response.

Materials & experimental systems

n/a	Involved in the study	
☒	☐	Antibodies
☒	☐	Eukaryotic cell lines
☒	☐	Palaeontology and archaeology
☒	☐	Animals and other organisms
☒	☐	Clinical data
☒	☐	Dual use research of concern

Methods

n/a	Involved in the study	
☒	☐	ChIP-seq
☒	☐	Flow cytometry
☒	☐	MRI-based neuroimaging
